# Efficacy and safety of collagenase clostridium histolyticum for Dupuytren disease nodules: a randomized controlled trial

**DOI:** 10.1186/s12891-017-1713-z

**Published:** 2017-08-30

**Authors:** Bronier Costas, Stephen Coleman, Greg Kaufman, Robert James, Brian Cohen, R. Glenn Gaston

**Affiliations:** 1 The Hand and Upper Extremity Center of Georgia, 980 Johnson Ferry Rd, NE, Suite 1020, Atlanta, GA 30342 USA; 2 Brisbane Hand and Upper Limb Clinic, 259 Wickham Terrace, Spring Hill, Queensland, 4000 Australia; 3 Auxilium Pharmaceuticals, Inc, 640 Lee Rd, Chesterbrook, PA 19087 USA; 4 OrthoCarolina, 1915 Randolph Rd, Charlotte, NC 28207 USA

**Keywords:** Clostridium, Dupuytren contracture, Hand, Microbial collagenase, Xiaflex

## Abstract

**Background:**

To determine the safety and efficacy of collagenase clostridium histolyticum (CCH) injection for the treatment of palmar Dupuytren disease nodules.

**Methods:**

In this 8-week, double-blind trial, palpable palmar nodules on one hand of adults with Dupuytren disease were selected for treatment. Patients were randomly assigned using an interactive web response system to receive a dose of 0.25 mg, 0.40 mg, or 0.60 mg (1:1:1 ratio) and then allocated to active treatment (CCH) or placebo (4:1 ratio). All patients and investigators were blinded to treatment. One injection was made in the selected nodule on Day 1. Caliper measurements of nodule length and width were performed at screening and at Weeks 4 and 8. Investigator-reported nodular consistency and hardness were evaluated at baseline and Weeks 1, 4, and 8. Investigator-rated patient improvement (1 [very much improved] to 7 [very much worse]) and patient satisfaction were assessed at study end.

**Results:**

In the efficacy population (*n* = 74), percentage changes in area were significantly greater with CCH 0.40 mg (−80.1%, *P* = 0.0002) and CCH 0.60 mg (−78.2%, *P* = 0.0003), but not CCH 0.25 mg (−58.3%, *P* = 0.079), versus placebo (−42.2%) at post-treatment Week 8. Mean change in nodular consistency and hardness were significantly improved with CCH versus placebo at Weeks 4 and 8 (*P* ≤ 0.0139 for all). At Week 8, investigator global assessment of improvement was significantly greater with CCH 0.40 mg and 0.60 mg (*P* ≤ 0.0014) but not statistically significant with CCH 0.25 mg versus placebo (*P* = 0.13). Most patients were “very satisfied” or “quite satisfied” with CCH 0.40 mg and 0.60 mg. Contusion/bruising (50.0% to 59.1%) was the most common adverse event with CCH treatment.

**Conclusion:**

In patients with Dupuytren disease, a single CCH injection significantly improved palmar nodule size and hardness. The safety of CCH was similar to that observed previously in patients with Dupuytren contracture.

**Trial registration:**

ClinicalTrials.gov identifier: NCT02193828. Date of trial registration: July 2, 2014 to December 5, 2014

## Background

Dupuytren disease is a common fibroproliferative disease of the palmar fascia [1] that is reported to affect between 1% and 32% of individuals in Western countries [2, 3]. It is characterized by the formation of thick collagen nodules that can progress to fibrous cords capable of producing digital flexion contractures and reducing hand function [4]. Dupuytren disease exhibits three clinical phases known as the proliferative, contractile, and residual phases [5]. In the early proliferative phase, nodules form as myofibroblasts and proliferate around microvessels [5]. This myofibroblast proliferation may lead to vessel occlusion and hypoxia, and signal infiltration of immune cells [5]. Expression of inflammatory signals and growth factors (eg, transforming growth factor-β) by immune cells may stimulate myofibroblast differentiation [6] and contraction [7] and augment the production of extracellular matrix proteins, such as fibronectin and collagen within nodules [8, 9]. In the contractile phase, nodules are reduced in size and myofibroblasts become arranged around the major areas of stress within the nodule, forming a cord [4]. Myofibroblasts also continue to produce collagen, particularly Type III, as well as fibronectin [4]. In the residual phase, nodules have been replaced by fibrous cords, which can shorten and cause further contracture [4].

Currently, no treatment has been approved for nodules associated with Dupuytren disease, although many nodules are symptomatic when pressure is applied to the palm and many will progress to cords with resultant contracture [10]. When treatment (eg, the injection of collagenase clostridium histolyticum [CCH] or surgery) is considered appropriate, it is generally applied during the contractile and residual phases once cords have developed. However, given that collagen augments the disease process and decreases with disease progression [4, 11, 12], earlier treatment with agents that disrupt collagen formation (eg, CCH) is thought to potentially alter disease progression and reduce nodule size, symptoms, and clinical impact [13, 14].

The CCH formulation Xiaflex® (Endo Pharmaceuticals Inc., Malvern, PA, USA) is a combination of two *Clostridium histolyticum* collagenases (AUX-I and AUX-II) that is currently approved in the United States, Europe, and Australia for the treatment of adult patients with Dupuytren contracture with a palpable cord [1]. These enzymes hydrolyze type I and type III collagen into smaller peptides, which may then be degraded by endogenous human collagenases [1]. In two phase 3 trials (Collagenase Option for the Reduction of Dupuytren [CORD I and CORD II]), injection of CCH into the cords of patients with Dupuytren contracture reduced joint contraction to 0–5° of full extension within 30 days of the last injection in a significantly greater percentage of joints versus placebo injection (CORD I: 64.0% with CCH vs 6.8% with placebo; CORD II: 44.4% vs 4.8%; *P* < 0.001 for both) [13, 14]. This phase 2a study evaluated the safety and efficacy of multiple doses of CCH injections for the treatment of palmar Dupuytren disease nodules.

## Methods

### Patient population

Patients ≥18 years of age with Dupuytren disease who had ≥1 palpable palmar nodule that was not associated with a cord and measured between 0.5 cm and 2.0 cm in length and between 0.5 cm and 2.0 cm in width were eligible for inclusion in the study. Patients who had received steroid injections or collagenase treatment (including Santyl® ointment, Smith & Nephew, Inc., Fort Worth, TX, USA) for the treatment of the selected nodule within the past 30 days or surgery on the selected hand within 3 months were excluded. Patients were also ineligible if they had a chronic hand-related muscular, neurologic, or neuromuscular condition, had received or were planning to receive anticoagulant medication within 7 days of study initiation, or had a recent history of stroke or bleeding. All patients included in the study received injection of either CCH or placebo.

### Clinical study design

This 8-week, double-blind, placebo-controlled, exploratory phase 2a study (ClinicalTrials.gov identifier: NCT02193828) was conducted between July 2, 2014, and December 5, 2014 at 11 centers in the United States and Australia. During the screening visit, a palpable palmar nodule on one hand was selected to receive treatment for each eligible patient. On Day 1, patients were randomly assigned to a dose group (based on doses of CCH evaluated in the study) in a 1:1:1 ratio and then further randomly assigned to active treatment [CCH] or placebo in a 4:1 ratio using an interactive web response system. All patients and study site personnel involved in patient evaluation, including the investigators, were blinded to treatment throughout the study. Both CCH and placebo were reconstituted in a solution containing 0.9% NaCl and 0.03% CaCl. Patients received CCH 0.25 mg, 0.40 mg, or 0.60 mg (plus Tris-HCl and sucrose) in a 0.11-, 0.17-, or 0.21-mL total injection volume, respectively, or volume-matched placebo (Tris-HCl and sucrose). Different injection volumes for each treatment group were necessary to ensure delivery of the appropriate concentration of CCH. Patients received a single injection directly into the selected hand nodule using a 26- or 27-gauge, 13-mm needle. The needle was inserted horizontally along the length of the nodule but did not penetrate the opposite side of the nodule. Treatment volume was dispensed as the needle was withdrawn to ensure complete deposition within the nodule.

Patients were monitored for immediate immunologic adverse events (AEs) for 20 min post-injection. Follow-up visits occurred at post-injection Week 1, Week 4, and Week 8. Starting at Week 1, all patients were instructed to massage the nodule (massage for 30 s, rest for 30 s, and repeat) twice daily until Week 4. The study was approved by central or local institutional review boards at each participating center within Australia and the United States and followed Good Clinical Practice and principles expressed in the Declaration of Helsinki. All patients provided written informed consent.

### Study assessments

The size of the selected nodule was measured at screening, Week 4, and Week 8 using hand-held calipers (for length and width), and at screening and Week 8 using ultrasonography (for length, width, and depth). Nodular consistency was rated by the investigator on a 5-point scale (5 [hard/solid], 4 [firm throughout], 3 [moderate firmness], 2 [soft], or 1 [non-palpable]) after palpation of the selected nodule on Day 1 (the day of injection) and at Weeks 1, 4 and 8. Nodule hardness and pain were assessed on Day 1 and at Weeks 1, 4 and 8. A durometer was used to assess the hardness of the selected nodule with a range of 0–100. Nodular pain was induced using a dynamometer (by applying direct pressure to the nodule) and was then measured on a visual analog scale from 0 (no pain or discomfort) to 10 (extreme pain or discomfort). Investigators rated patient improvement from screening to Week 8 on a scale from 1 (very much improved) to 7 (very much worse). Patient satisfaction with treatment was assessed at Week 8 using a 5-point scale: 1 (very satisfied), 2 (quite satisfied), 3 (neither satisfied nor dissatisfied), 4 (quite dissatisfied), and 5 (very dissatisfied). Treatment-emergent AEs were monitored and vital signs were collected throughout the study. Serum samples for the determination of AUX-I and AUX-II antibodies were collected at screening and the final visit (Week 8).

### Statistical analysis

Sample size was estimated assuming response rates of 15% for all placebo groups, 50% for CCH 0.60-mg, 40% for CCH 0.40-mg, and 35% for CCH 0.25-mg groups. Thus, a sample size of 80 patients would be required to achieve ≥85% power to detect differences between placebo and CCH 0.60 mg or CCH 0.40 mg. Assuming a common dropout rate (10%), 90 patients were determined to be sufficient for enrollment.

The safety population included any patients who received an injection of the study drug. All patients in the safety population who also had pre- and post-injection nodule measurements were included in the efficacy population. The primary end point was the percentage change from baseline in surface area, as measured by calipers, of the treated nodule at Week 8. Secondary end points included percent change from baseline in surface area (as measured by ultrasound) of the treated nodule at Week 8, change from baseline in consistency and hardness of the treated nodule at Week 8, change in nodule pain from baseline to Week 8, investigator global assessment of improvement and patient satisfaction at Week 8, and composite responder analysis at Week 8. Patients who reported being satisfied with treatment (ie, responded very satisfied [1] or quite satisfied [2]) and reported improvement according to investigator assessment (ie, very much improved [1], much improved [2], or minimally improved [3]) were considered composite responders.

Between-group differences in categorical variables other than the composite responder end point (ie, investigator global assessment of improvement, patient satisfaction, nodular consistency, change from baseline in nodular consistency) were analyzed using a Kruskal-Wallis test. Differences between each CCH-dose group and placebo were compared using a Mann-Whitney test. For the composite responder end point, the Fisher’s exact test was used to analyze between-group comparisons. One-way analysis of variance was used to assess between-group differences in continuous variables (percent change in area [using caliper or ultrasound measurement], nodular hardness, and change in nodular pain). Pairwise comparisons were performed to compare each CCH dose and placebo.

Occurrences of AEs were reported using descriptive statistics. The overall count and percentage of patients with AUX-I and AUX-II antibodies were summarized as categorical variables. Log-transformed AUX-I and AUX-II titer values and vital sign measurements were summarized as continuous variables.

## Results

### Study population

**Fig. 1:**
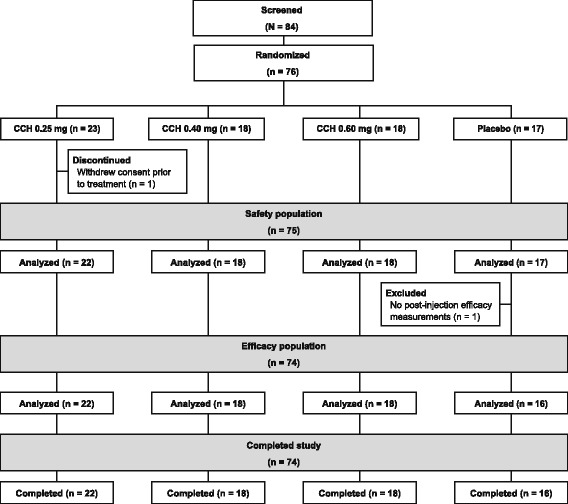
Patient disposition. One patient withdrew consent before receiving study drug on Day 1 and was excluded from all analyses (safety and efficacy). CCH, collagenase clostridium histolyticum

Of 84 patients screened, 76 patients met eligibility criteria and were randomly assigned to treatment. Of those, 75 patients were included in the safety population (1 patient withdrew consent before treatment administration; Fig. 1). Demographics and baseline characteristics for the safety population (*n* = 75) were similar among groups (Table 1). Seventy patients overall (86.4% to 100.0% of patients in each treatment group) had not received previous treatment for Dupuytren disease. One patient in the safety population received study medication but did not complete any post-treatment efficacy evaluations; therefore, only 74 patients were included in the efficacy analyses (Fig. 1).

**Table 1:** Demographic and Baseline Characteristics^a^

Parameter	CCH 0.25 mg (*n* = 22)	CCH 0.40 mg (*n* = 18)	CCH 0.60 mg (*n* = 18)	Placebo (*n* = 17)
Mean age, y (SD)	57.9 (10.0)	58.1 (12.4)	60.0 (10.2)	59.9 (8.8)
Sex, n (%)				
Female	11 (50.0)	10 (55.6)	6 (33.3)	7 (41.2)
Male	11 (50.0)	8 (44.4)	12 (66.7)	10 (58.8)
Race, n (%)				
White	22 (100.0)	17 (94.4)	18 (100.0)	17 (100.0)
Other	0	1 (5.6)	0	0
Mean age at Dupuytren disease onset, y (SD)	51.6 (13.5)	50.4 (13.8)	55.8 (8.3)	54.7 (11.2)
Nodules on selected hand, n (%)				
1	9 (40.9)	9 (50.0)	10 (55.6)	6 (35.3)
2	6 (27.3)	3 (16.7)	4 (22.2)	5 (29.4)
≥ 3	7 (31.8)	6 (33.3)	4 (22.2)	6 (35.3)
Mean nodule area^b^, cm^2^ (SD)	0.7 (0.3)	0.7 (0.4)	0.7 (0.3)	0.8 (0.4)
Prior Dupuytren disease treatments				
None	19 (86.4)	18 (100.0)	16 (88.9)	17 (100.0)
Fasciectomy	2 (9.1)	0	0	0
Needle aponeurotomy	0	0	1 (5.6)	0
CCH	1 (4.5)	0	2 (11.1)	0

^a^Safety population (*n* = 75)

^b^Measured using calipers. Calculated as 0.79 × length × width

*CCH* collagenase clostridium histolyticum, *SD* standard deviation

### Efficacy

**Fig. 2:**
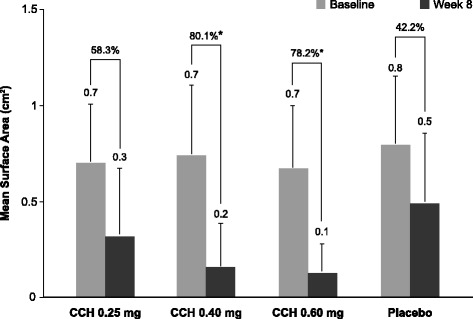
Nodular area at baseline and Week 8. Error bars represent standard deviations. ^*^ *P* ≤ 0.0003 vs placebo. CCH, collagenase clostridium histolyticum

**Fig. 3:**
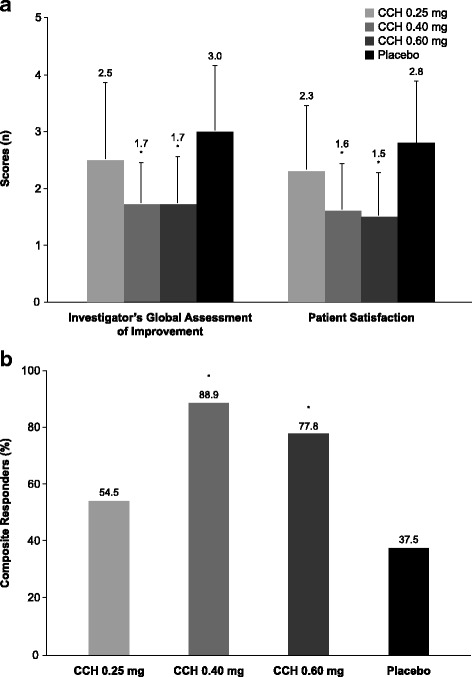
Investigator- and patient-reported assessments at Week 8. Investigator-reported improvement (rating: 1 [very much improved] to 7 [very much worse]) and patient-reported satisfaction (rating: 1 [very satisfied] to 5 [very dissatisfied]) (**a**) and percentage of composite responders (**b**). Error bars represent standard deviations. ^*^ *P* ≤ 0.03 vs placebo. CCH, collagenase clostridium histolyticum

In the efficacy population at Week 4, improvements in caliper-measured nodular surface area (change from baseline: CCH 0.25 mg, −0.30 cm^2^; CCH 0.40 mg, −0.49 cm^2^; CCH 0.60 mg, −0.50 cm^2^) were numerically greater in all CCH groups versus placebo (−0.21 cm^2^). Percentage reductions in area at Week 4 were significantly greater with CCH 0.40 mg (−58.8%, *P* = 0.0109) and CCH 0.60 mg (−72.4%, *P* = 0.0003) versus placebo (−27.9%), but not with CCH 0.25 mg (−41.4%; *P* = 0.24). At Week 8, significant differences versus placebo were observed in caliper-measured nodular surface area for CCH 0.60 mg (*P* = 0.0003) and CCH 0.40 mg (*P* = 0.0002), but not with CCH 0.25 mg (*P* = 0.08; Fig. 2) Ultrasound measurements of nodule size did not correlate with direct caliper measurements and were, therefore, considered an unreliable assessment of treatment efficacy and not reported for this study. Nodular consistency and hardness improved from baseline to Week 1, with significant improvements in all CCH groups versus placebo at Weeks 4 and 8 (Table 2). At Week 8, soft or non-palpable nodules were observed in 8 (36.4%) of 22 nodules in the 0.25-mg group, 12 (70.6%) of 17 nodules in the CCH 0.40-mg group, and 12 (75.0%) of 16 nodules in the CCH 0.60-mg group. Baseline median pain scores were low for all treatment groups (placebo and CCH 0.25 mg [2.0], CCH 0.40 mg [0.5], CCH 0.60 mg [0.0]), illustrating that most patients had little to no nodular pain at study initiation. Significant improvement in nodular pain from baseline was not observed between any CCH group and placebo at any time point. Investigator global assessment of improvement and patient satisfaction at Week 8 were significantly greater in the 0.60-mg and 0.40-mg CCH groups versus placebo (Fig. 3a). A significantly greater percentage of patients in the higher CCH-dose groups were composite responders (CCH 0.40 mg, 88.9%, *P* = 0.003; CCH 0.60 mg, 77.8%, *P* = 0.03) compared with those in the placebo group (37.5%; Fig. 3b). Although the percentage of responders in the 0.25-mg group (54.5%) was numerically greater than that reported for placebo responders (37.5%), this difference was not statistically significant (*P* = 0.34).

**Table 2:** Nodule Consistency and Hardness

Parameter	CCH 0.25 mg (*n* = 22)	CCH 0.40 mg (*n* = 18)	CCH 0.60 mg (*n* = 18)	Placebo (*n* = 16)
Nodule Consistency Score^a^				
Baseline, mean (SD)	4.2 (0.7)	4.1 (0.7)	4.1 (0.5)	3.7 (0.6)
Week 1^b^				
Mean (SD)	3.1 (0.8)	2.7 (0.8)	2.6 (0.8)	3.6 (0.7)
Mean change from baseline (SD)	−1.0 (0.8)	−1.4 (0.6)	−1.4 (0.9)	−0.1 (0.7)
Week 4				
Mean (SD)	3.1 (0.8)	2.4 (1.0)^c^	2.0 (0.8)^d^	3.5 (0.8)
Mean change from baseline (SD)	−1.1 (0.9)^c^	−1.7 (0.8)^d^	−2.1 (0.9)^d^	−0.2 (0.8)
Week 8				
Mean (SD)	3.0 (1.1)	2.2 (1.0)^c^	2.1 (0.8)^d^	3.4 (1.0)
Mean change from baseline (SD)	−1.2 (1.1)^c^	−1.9 (1.1)^d^	−1.9 (0.9)^d^	−0.3 (1.0)
Nodule Hardness Score^e^				
Baseline, mean (SD)	68.7 (12.5)	67.0 (8.8)	68.2 (8.0)	63.0 (10.0)
Week 1^b,f^				
Mean (SD)	58.3 (12.8)	52.8 (8.6)	55.0 (10.4)	65.3 (10.6)
Mean change from baseline (SD)	−10.4 (13.3)	−14.2 (12.5)	−13.2 (11.8)	2.3 (12.6)
Week 4^g^				
Mean (SD)	56.4 (10.9)	54.7 (9.3)	55.6 (12.6)	63.1 (11.6)
Mean change from baseline (SD)	−12.0^c^ (11.3)	−12.3 (10.6)^c^	−13.1 (14.3)^c^	0.3 (12.6)
Week 8^h^				
Mean (SD)	55.9 (15.2)	46.9 (17.8)	56.4 (10.9)	64.3 (10.4)
Mean change from baseline (SD)	−12.8 (14.9)^c^	−19.6 (14.4)^d^	−12.1 (11.8)^c^	1.5 (12.5)

^a^Nodular consistency was rated as 5 (hard/solid), 4 (firm throughout), 3 (moderate firmness), 2 (soft), or 1 (non-palpable). Negative percentage change indicates improvement

^b^Statistical analyses were not performed on Week 1 data

^c^ *P* < 0.02 vs placebo

^d^ *P* < 0.001 vs placebo

^e^Hardness of the nodule was assessed using a durometer on a scale of 0–100

^f^Placebo, *n* = 16; CCH 0.25 mg, *n* = 22; CCH 0.40 mg, *n* = 18; CCH 0.60 mg, *n* = 18

^g^Placebo, *n* = 15; CCH 0.25 mg, *n* = 21; CCH 0.40 mg, *n* = 18; CCH 0.60 mg, *n* = 17

^h^Placebo, *n* = 15; CCH 0.25 mg, *n* = 22; CCH 0.40 mg, *n* = 17; CCH 0.60 mg, *n* = 16

*CCH* collagenase clostridium histolyticum, *SD* standard deviation

### Safety

The most common AEs in the CCH groups were contusion/bruising, extremity pain, and localized swelling (Table 3). There were no trends for increased AE occurrence with increasing CCH dose, except for injection-site bruising and localized swelling. Most AEs in all CCH groups were mild (84.5% with 0.25 mg, 69.1% with 0.40 mg, and 84.2% with 0.60 mg) or moderate (15.5%, 30.9%, 14.0%, with CCH 0.25 mg, 0.40 mg, and 0.60 mg, respectively). Severe treatment-related injection-site pain was reported in one patient receiving CCH 0.60 mg. No clinically meaningful changes in vital signs were observed. No deaths or patient discontinuations because of a treatment-emergent AE were reported. At Week 8, most patients in all CCH groups (86.4–100.0%) tested positive for antibodies against AUX-I and AUX-II; however, mean log antibody titers were low (ie, <3.2).

**Table 3:** Adverse Events Reported by ≥2 Patients in Any Treatment Group (Safety Population)^a^

AE, n (%)	CCH 0.25 mg (*n* = 22)	CCH 0.40 mg (*n* = 18)	CCH 0.60 mg (*n* = 18)	Placebo (*n* = 17)
Any AE	21 (95.5)	18 (100.0)	17 (94.4)	7 (41.2)
Discontinuations due to AEs	0	0	0	0
Any serious AE	0	0	0	0
Contusion/bruising	13 (59.1)	9 (50.0)	9 (50.0)	1 (5.9)
Extremity pain	10 (45.5)	10 (55.6)	7 (38.9)	1 (5.9)
Local swelling	8 (36.4)	7 (38.9)	10 (55.6)	3 (17.6)
Injection-site bruising	5 (22.7)	4 (22.2)	6 (33.3)	0
Axillary pain	6 (27.3)	1 (5.6)	4 (22.2)	0
Injection-site pain	4 (18.2)	4 (22.2)	2 (11.1)	0
Injection-site swelling	5 (22.7)	4 (22.2)	0	0
Injection-site pruritus	2 (9.1)	3 (16.7)	2 (11.1)	1 (5.9)
Injection-site edema	2 (9.1)	0	2 (11.1)	0
Pruritus	2 (9.1)	2 (11.1)	1 (5.6)	0
Injection-site hemorrhage	2 (9.1)	0	1 (5.6)	0

^a^Presented in order of occurrence in the active treatment groups

*AE* adverse event, *CCH* collagenase clostridium histolyticum

## Discussion

Currently, no treatments have been approved for Dupuytren nodules, although a retrospective chart review by Reilly et al. showed that 51% of patients with nodules who returned for follow-up (mean time between diagnosis and follow-up: 8.7 years, range, 6–15 years) had developed a cord and 8% had progressed to full contracture [10]. In addition, nodules may be painful in some patients and impair their ability to grip objects or use their hands successfully. Although the pathophysiology underlying Dupuytren disease remains a controversial topic, inflammatory and growth factor signals likely play a role through the augmentation of specific aspects of the disease (eg, myoblast proliferation and collagen production) [5, 6, 8, 9]. Dupuytren nodules are rich in collagen type I and III (ie, the substrates for CCH) [15] and in vitro, CCH has been shown to reduce the expression of extracellular matrix components, cytokines, and growth factors that may contribute to nodule formation and progression [15]. Thus, the properties of CCH at the site of local injection suggest CCH as a possible treatment option for nodules.

The results of the current phase 2a, dose-ranging study support continued investigation into the efficacy and safety of CCH for the treatment of Dupuytren nodules. Despite a greater than expected improvement in caliper-measured nodular surface area from baseline to Week 8 in the placebo group (42.2%), improvement was only significantly greater with CCH 0.40 mg (80.1%, *P* = 0.0002) and 0.60 mg (78.2%, *P* = 0.0003). Improvement in the lowest CCH-dose group (0.25 mg: 58.3%) was numerically greater than that observed with placebo (42.2%); however, the difference did not reach statistical significance (ie, *P* > 0.05). Significant improvements from baseline versus placebo were observed in the CCH 0.25-mg group for nodule hardness and consistency. However, greater improvement was observed at the two higher CCH doses (0.40 mg and 0.60 mg), with little apparent increase in the incidence of AEs. Furthermore, investigators noted “very much” or “much” improvement in most (83.3% with CCH 0.40 mg and 88.9% with CCH 0.60 mg) patients who received the two higher doses of CCH. Most patients also expressed a high degree of satisfaction with CCH treatment, indicating that they were “very satisfied” or “quite satisfied” with the two higher CCH doses. Based on these data, CCH doses greater than 0.25 mg appear to be more effective than lower doses for the treatment of Dupuytren nodules and warrant further investigation.

Clinical trials have demonstrated the beneficial effect of CCH for the treatment of Dupuytren contracture [13, 14]. During these trials, joints with low baseline contracture severity had greater reduction in contracture to 0–5° of normal (primary end point) 30 days post-injection than joints with more severe contracture [13, 14], implying that earlier treatment may have an effect on the potential response to CCH. However, the current medical literature for the pharmacologic treatment of Dupuytren nodules is limited. In a 4-year study of patients with Dupuytren nodules (*n* = 75 hands), injection of triamcinolone acetonide (a corticosteroid) flattened and softened the injected nodules in most (97%) hands. However, multiple injections per site were performed (mean number of injections, 3.2), and the authors concluded that the initial injection of corticosteroids was more of a “priming” than a therapeutic dose [16]. The current study demonstrated that injection of CCH into nodules significantly improved nodule consistency and reduced hardness versus placebo within 4 weeks after a single injection.

The overall safety profile of CCH was similar to that reported in phase 3 clinical trials of CCH for treatment of Dupuytren contracture [13, 14]. The most commonly reported AEs (ie, contusion/bruising, extremity pain, and local swelling) with the injection of CCH into nodules were similar to those previously reported with CCH injection for the treatment of Dupuytren contracture [13, 14]. Most patients (86.4–100.0%) had antibodies against AUX-I and AUX-II, which was consistent with the rate reported for patients receiving injection into a Dupuytren cord (82–95.2%) [13, 14]. Research has also shown that the presence of AUX-I and AUX-II antibodies has no impact on the efficacy or safety of later injections [17–19].

The current study is limited by its small sample size per treatment group, the administration of only one injection, the limited follow-up duration, and an inability to quantify changes in nodules accurately using ultrasound. Discordance between caliper and ultrasound measurements of nodule size was related to extreme outliers and lack of convergent validity with other efficacy measures. This was likely because of a lack of existing standards for use of ultrasound to measure nodules. Similar patterns of results were observed for both caliper and ultrasound measurements, with the CCH 0.40-mg and 0.60-mg groups showing greater reduction in nodule size compared with placebo, but the wide variability in the ultrasound measurements prevented computation of any significant treatment effect. Thus, we recommend that standard measurement rules be pre-specified in future studies using ultrasound measurements of nodules, and that personnel conducting ultrasound assessments undergo training to maximize measurement consistency. Some improvement was noted in the placebo group for all efficacy end points, which suggests that factors other than active treatment (eg, local injection of anesthesia, nodular massage alone, or patient expectation [placebo response effect]) may have impacted the results. However, the fact that significant improvements with CCH treatment were observed despite the high placebo rate may indicate that benefits of CCH are potentially greater than what has been reported in the current study. In addition, ratings of nodule consistency, nodule pain, and patient satisfaction were subjective; and although both the patient and investigators were blinded to treatment, it is possible that these end points were affected by individuals’ desire for or anticipation of improvement. However, the consistency of the improvement observed among all subjective and non-subjective assessments (eg, nodule size as measured with calipers and durometer measurements of hardness) suggests the subjective measurements used in the current study accurately assessed an effect of treatment. Finally, practical use of a dynamometer to potentiate pressure on the affected nodule and then measure nodule pain had not been previously studied in this type of clinical scenario with Dupuytren disease. The positioning of the dynamometer against the nodule was not standardized; thus, patients may not have applied direct pressure to the nodule if it was painful. This variation to avoid pain may explain why no significant improvements in pain were observed with CCH versus placebo. Despite these issues, CCH treatment improved nodular pain by the end of the study and a treatment effect was observed in a post hoc analysis of patients with baseline pain scores ≥3.

## Conclusion

This phase 2a, dose-ranging study demonstrated that a single injection of CCH 0.40 mg or 0.60 mg significantly decreased the size and hardness of palmar nodules in patients with Dupuytren disease and displayed a tolerable safety profile, similar to that reported with CCH treatment for Dupuytren contracture. Additional studies are needed to confirm these initial results and evaluate the long-term efficacy and safety of CCH for palmar nodules.

## Abbreviations

AE: adverse event
CCH: collagenase clostridium histolyticum
CORD: Collagenase Option for the Reduction of Dupuytren
